# Moving Object Tracking Based on Sparse Optical Flow with Moving Window and Target Estimator

**DOI:** 10.3390/s22082878

**Published:** 2022-04-08

**Authors:** Hosik Choi, Byungmun Kang, DaeEun Kim

**Affiliations:** School of Electrical and Electronic Engineering, Yonsei University, Seoul 03722, Korea; hosikchoi@yonsei.ac.kr (H.C.); kbmang@yonsei.ac.kr (B.K.)

**Keywords:** moving object tracking, optical flow, moving window, target estimator

## Abstract

Moving object detection and tracking are technologies applied to wide research fields including traffic monitoring and recognition of workers in surrounding heavy equipment environments. However, the conventional moving object detection methods have faced many problems such as much computing time, image noises, and disappearance of targets due to obstacles. In this paper, we introduce a new moving object detection and tracking algorithm based on the sparse optical flow for reducing computing time, removing noises and estimating the target efficiently. The developed algorithm maintains a variety of corner features with refreshed corner features, and the moving window detector is proposed to determine the feature points for tracking, based on the location history of the points. The performance of detecting moving objects is greatly improved through the moving window detector and the continuous target estimation. The memory-based estimator provides the capability to recall the location of corner features for a period of time, and it has an effect of tracking targets obscured by obstacles. The suggested approach was applied to real environments including various illumination (indoor and outdoor) conditions, a number of moving objects and obstacles, and the performance was evaluated on an embedded board (Raspberry pi4). The experimental results show that the proposed method maintains a high FPS (frame per seconds) and improves the accuracy performance, compared with the conventional optical flow methods and vision approaches such as Haar-like and Hog methods.

## 1. Introduction

Technologies to detect and track moving objects are of significance in many applications such as unmanned vehicles and surveillance cameras to detect and recognize pedestrians and track workers in factory environments to ensure personnel safety. Conventional algorithms to detect and track moving objects include frame difference algorithms, background subtraction algorithms, optical flow-based algorithms, and static learning algorithms. In addition, various deep-learning-based moving-object detection algorithms based on deep convolution networks such as YOLOv5s and R-CNNs have been developed. However, these methods are highly sensitive to changes in the background brightness, which increases the probability of erroneous detection. Furthermore, deep-learning-based algorithms are computationally complex, require sufficient training samples, and are not suitable for real-time processing on a board without graphical processing units (GPUs). In addition, conventional moving-object detection and tracking algorithms cannot effectively detect and track targets obscured by obstacles within the images or when an image is distorted by camera vibrations. These limitations are particularly concerning, especially in applications aimed at ensuring the safety of workers operating near heavy equipment. The Korea Occupational Safety and Health Agency (KOSHA) reports that among the many causes of safety-related accidents, the most notable cause of collisions with objects around heavy equipment in workplaces is the lack of visibility of equipment workers in the environment.

To address this problem, this paper proposes a moving-object detection and tracking algorithm that can be applied to various safety-related applications. The proposed algorithm can efficiently eliminate the camera vibration related noise in the image frames and perform continuous tracking for moving objects obscured by obstacles. Specifically, the conventional sparse optical flow algorithm (Lucas-Kanade, LK) is enhanced to detect and track multiple moving objects at a low computational cost. Moreover, the corner extraction algorithm (Shi–Tomasi) is used to track feature points to detect and track moving targets. A moving window detector and memorized estimator are used to enhance the detection performance, ensure the robustness of the algorithm to noise, and improve the worker safety. In particular, the moving window detector uses the window memory at each feature point as the window size and detects and tracks the moving target by evaluating whether the feature point is noise or a moving object. The location history of the detected points is memorized, and a halted or invisible target is identified from the location history of the feature points. Subsequently, the estimator decides whether the target state is maintained.

1. The proposed moving-object detection algorithm is computationally effective, and the performance of the sparse optical flow algorithm based on the LK method is enhanced using the Shi–Tomasi corner extraction algorithm. Moreover, a novel moving window detector and memorized estimator function are used.
2. The performance is enhanced by operating on a non-GPU platform and suing a low computational power embedded system such as Raspberry Pi.
3. The moving window detector helps enhance the robustness of the proposed approach against vibration related noise in surveillance system environments.
4. The memorized estimator function can prevent accidents of workers in fields with hazards and obstacles.

Section 2 describes the existing studies related to moving-object detection and tracking algorithms. Section 3 presents an overview of the three processes of the proposed algorithm and describes the dataset. Section 4 describes the determination of the parameters of the proposed algorithm and comparison of the experimental results with those obtained using existing algorithms. Section 5 describes the limitations and scope for future work.

## 2. Related Works

In recent decades, methods to detect and track moving objects have been widely applied. Before the development of learning-based algorithms, methods based on the optical flow, frame difference, and background subtraction were typically used. In these algorithms, the difference between frames was used to determine the movement of objects, and thus, the objects could be accurately detected. However, these algorithms were computationally intensive and complex.

Certain researchers attempted to perform object recognition using the optical flow based on a camera attached to a moving vehicle [1]. Several movements were captured within the scene, and the ego motion was separated from the background. However, when the scene moved instead of a fixed camera, many false positives occurred. The authors attempted to relax the stationary cameras restriction by using traditional moving-object detection methods and introducing additional steps before and after the detection. For cameras to be attached to heavy equipment, a fisheye camera with a wide-angle range can be used. Certain researchers developed an approach to track pedestrians and cars in fisheye images [2], using low-cost sensors and four fisheye cameras with a wide range. An unwarping technique is used to pre-process distorted images, followed by object classification and tracking. A novel equipment design and sensing system (Safety 360) was developed to provide equipment operators with a surround-view [3]. Moreover, various optical flow techniques for moving-object detection have been proposed [4]. Moving-object tracking was realized using optical flow and motion vector estimation [5,6], and the approach was noted to exhibit a strong object tracking ability for the same scene in various views. To perform real-time object detection and tracking, feature extraction was conducted using the pyramid LK optical flow, as a sparse optical flow technique [7]. To enhance the tracking accuracy, the corners were detected for tracking, the subpixel corners were determined, the video in each frame of the image layered in the image pyramid was examined to calculate the optical flow at the top corner, and the next pyramid was considered the starting point of the pyramid. This process was repeated until the bottom pyramid image. Notably, object recognition can be supplemented in a moving camera situation with technological advancements.

Moreover, the object detection performance for a complex background can be enhanced using the optical flow. Object detection may not be effective when the frame difference technique is integrated with the optical flow technique. Certain researchers developed algorithms to perform background modelling tasks, using edge detection to solve problems [8]. Moreover, an object detection study was performed to clarify the influence of images distorted in environments such as those involving movement of the background, camera shaking, and rotation [9]. Moving-object detection was performed using images recorded at large distances, such as top views [10]. Images with camera equipment movement or shaking were pre-processed through background compensation, YOLOv3-SOD deep learning, and object detection.

Machine learning techniques have also been used to detect and recognize objects. The motion of Earth movement equipment was detected based on the vision at ground level [11]. Images were acquired from excavators or dump trucks, objects were tracked using the convolutional neural network (CNN) deep learning model, and routes were extracted using the hidden Markov model (HMM). Notably, the HMM leverages trajectories to train a Gaussian mixture model, and the probability density function of each activity can be determined using support vector machine (SVM) classifiers. For real-time vehicle detection and tracking for gas station surveillance, an approach based on the Adaboost classifier and optical flow tracking was proposed [12]. Specifically, the Adaboost algorithm was used to train the classifier with Haar-like features extracted from positive and negative samples of the gas station vehicles. Optical flow tracking method was performed to extract the corner points of the vehicle areas and match the positions of these corners in the consecutive frames in real-time.

Recently, many researchers developed approaches to detect moving objects by using optical flow and deep learning. With the widespread application of unmanned aerial vehicles (UAVs), moving objects have been attempted to be detected and tracked using cameras within the UAV [13]. In this approach, the moving objects were detected by subtracting a background changing in a complex manner from an image captured by a moving camera. The algorithm extracted motion areas based on optical flow and removed the background to perform clustering around moving objects. Noise was eliminated by removing a false foreground based on time and space consistencies. A frame skip strategy was used to accelerate the algorithm.

In addition, moving objects for UAVs were detected by obtaining images in real time. A dense optical flow technique was used; however, the background was assumed to be fixed. By obtaining top-view images in the aviation domain, the map for moving objects can be extracted using background removal and mean shift segmentation techniques. Notably, dense optical flow techniques are time intensive, and nearly 3.5 s are required per frame, which limits the application of such techniques to heavy equipment such as microcontroller units (MCUs) [14]. To address this limitation, we use a sparse optical flow technique. For UAVs, certain researchers proposed a robust onboard visual algorithm based on the reliable global-local object model for 2D and 3D object tracking to achieve a reasonable computational time [9]. This approach is based on global matching and local tracking. The algorithm initially identifies feature correspondences. An improved binary descriptor is developed for global feature matching, and an iterative LK optical flow algorithm is used for local feature tracking. Furthermore, an efficient local geometric filter is used to manage the outlier feature correspondences based on a new forward–backward pairwise dissimilarity measure, thereby ensuring pairwise geometric consistency. Section 3 in this paper describes the algorithm for eliminating noise via feature extraction. In another study, objects on the ground were identified using the YOLOv3 deep learning model for UAVs [15]. The images recorded by aircraft were transmitted to computers using in-flight communication systems, and neural network models were implemented through the computers.

Certain researchers developed a deep-learning-based framework for tracking UAVs. In this approach, moving objects (UAVs) were accurately detected at a high speed by modifying and improving CNN models based on YOLOv3-tiny in a real-time measured video stream [16]. The algorithm was characterized by multiple detection steps and tracking steps between frames. In the multiple detection phase, the FastUAV-NET architecture used five insertion units and a pyramid network. In the multi-tracking step, the detected boundary box was tracked using the scale-adaptive kernelized correlation filter (sKCF). Thus, algorithms to detect UAVs could be applied to every sixth frame, and efficient and accurate tracking could be performed in intermediate frames through sKCF [17]. This approach could effectively address the challenges associated with the high speed of UAVs, changes in the UAV scale and aspect ratio, variations in the illumination condition and camera viewpoint changes, and reflected light and shadows.

Certain researchers attempted to exploit consistent video frame information by directly applying image object detection technology to videos [18]. Notably, the direct application of image object detection models to video data is challenging owing to conditions such as motion blurring, video defocusing, and partial covering. Therefore, an algorithm was developed to accurately detect and track moving objects by readjusting the position of the bounding box by using the feature map of the target object of the key frame obtained based on YOLOv3 and the optical flow value of two adjacent frames obtained through FlowNET 2.0.

Moving-object detection techniques can also be applied for safety evaluations. Certain researchers used computer vision technologies to measure the vibration of buildings [19]. This approach could help evaluate the condition of the building, and minute movements of the building were detected using several sensors. The efficiency of the existing motion extraction methods was compared, using commercialized cameras and the LK optical flow instruments as experimental equipment. In general, when heavy equipment is operated and large vibrations and physical forces are applied to the ground at construction sites, shaking occurs throughout the structure. After pre-processing the image, this shaking can be monitored through monocular vision and detection of the obstacle around which the shaking occurs [20].

Object tracking algorithms have also been applied for ensuring personnel safety during the operation of heavy equipment such as unmanned excavators [21,22,23]. Motion detection and object tracking were performed using Velodyne VLP-16 light detection and ranging sensors. Moreover, motion predictions could be performed by analysing the physical movements and estimating the activity areas. Section 3 in this paper presents the proposed technique based on the optical flow [24,25,26,27,28]. Moreover, a stereo vision sensor-based monitoring system using more than one image can help distinguish various objects and represent them as three-dimensional information to ensure accurate monitoring [27,28]. Specifically, this technique can provide the three-dimensional geometry, high-resolution image correction, and colour and textural information to enhance the monitoring accuracy. However, for various lighting conditions, low resolution and high-performance camera systems may be required. Another approach can recognize and track objects by analysing the behaviour of workers at the construction site [29]. The moving objects are detected based on the optical flow, the joint probability around the detected objects is calculated using the naïve Bayesian model, and the workers’ actions are categorized to track and recognize the objects.

## 3. Proposed Method

**Figure 1:**
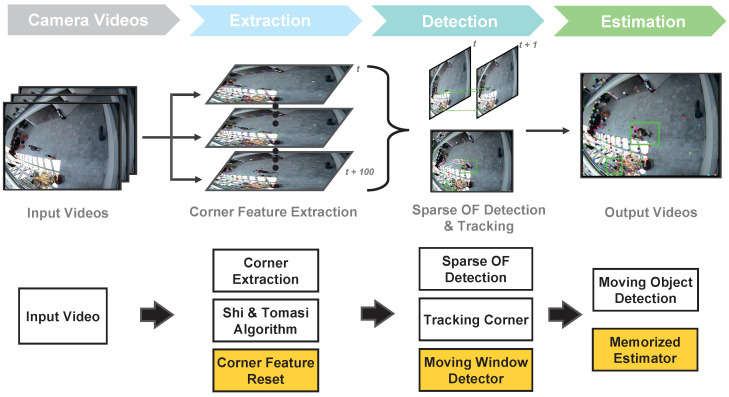
Overview of the proposed algorithm. We input videos with resolutions of 383 × 288 and 768 × 576. Every *N* frames, the corner feature points are generated with Shi-Tomasi algorithm [32]. The sparse optical flow algorithm calculates the moving information of feature points. The moving window detector collects the feature points for optical flow. If the tracking moving object disappears behind a wall, obstacles, or outside of a frame area, the memorized estimator checks the region around the target. The green boxes show detection of moving objects.

Figure 1 shows the process flow of the proposed real-time video-based algorithm. The proposed algorithm is based on sparse optical flow, which receives each frame of the video as the input and calculates the motion degree of the objects. Specifically, the algorithm calculates the motion information from all pixels of each frame, not as a dense optical flow (Farneback) [30] but as a sparse optical flow (pyramid LK) [31]. In addition, we extract the corner feature during LK optical flow calculation and estimate the moving object using only the moving information of this feature. More accurate and efficient motion information can be obtained by adding the regenerative function of the corner.

The LK method uses feature points to track the optical flow, which may render object detection over large distances challenging. In contrast to dense optical flow techniques, which evaluate all the pixels on the frame and neighbouring pixels, LK optical flow calculates feature points such as corners to facilitate tracking. Notably, if the moving objects are at a large distance, they are difficult to distinguish from noise. In detecting the motion information for each frame, a noise filtering function is introduced to alleviate the camera vibrations and light spread phenomena. To detect and track the moving objects obtained through feature extraction and LK optical flow, we introduce a memorized estimator to estimate the position of moving objects by memorizing information regarding the last missing position. This approach is expected to be effective in situations in which the object stops moving or is obscured by certain obstacles in the video input.

Notably, the proposed algorithm is based on the pyramid LK method but incorporates corner reset, noise filtering, and moving-object estimation (yellow box in Figure 1). Moreover, we aim to implement the proposed algorithm in embedded systems that can be used in places such as construction sites to ensure personnel safety.

### 3.1. Corner Feature Reset

For sparse optical flows, corner features are detected, and a set of features is used for optical flow calculation to decrease the computational time. The corner features are typically generated for a given image snapshot by using the Shi–Tomasi algorithm. Although moving objects can be tracked using optical flows, they may be hidden near obstacles or walls and appear again, or a new object may be observed in the image. Thus, corner feature reset must be performed to identify all the moving objects. Corner features are regenerated in regular period to prevent this problem. However, the process is not implemented for every frame because corner feature algorithms are time intensive.

**Figure 2:**
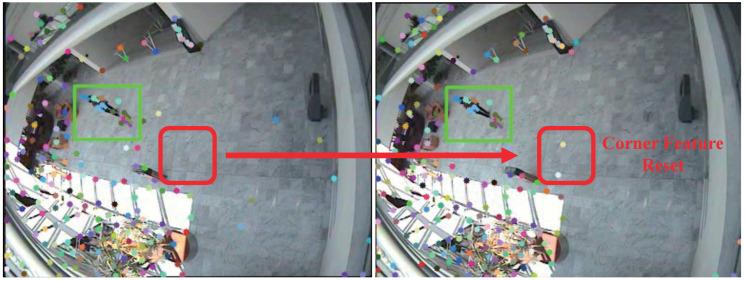
Example of the corner feature reset method. The red box shows that the corner feature reset method regenerates the corner features in a period of time. The green boxes show detection of moving objects.

Figure 2 shows an example of corner features based on the corner feature reset. The green box shows the detected moving object using proposed algorithm. The pedestrian features are retained for continuous tracking. The red box shows the region that the moving pedestrian traverses. This region may not be relevant for tracking anymore, but another moving object may be present in the area. Corner feature reset is aimed at identifying the corner features in the region for the optical flow of another moving object.

### 3.2. Moving Window Detector

**Figure 3:**
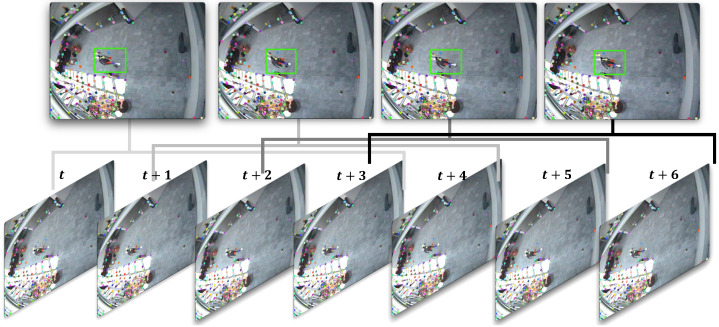
Example of the moving window detector method. The moving window method memorizes the feature points for optical flow within each of *n* frames. The green boxes show detection of a moving object.

The original pyramid LK method is vulnerable to noise such as that pertaining to light smudging in the input image and camera vibration. Even if no moving object is present in the actual optical flow, false positives may be induced owing to even small noises. To overcome these problems, we use a filtering function and enhance the detection performance in Figure 3.

(1) $$MW_{i,t}=\left\{P_{i,t-n},P_{i,t-n+1},\cdots ,P_{i,t-1}\right\}$$

The moving window function aims to memorize the feature points for optical flow within each of *n* frames. The presence of noise is evaluated by determining if the movement of the feature during an interval is less than or equal to the distance threshold. The window memory container $MW_{i,t}$ in Equation (1) continually saves the location of feature point *i* at time *t* over *M* points, $P_{i,t}$(i∈M). If t<n, the memory size is less than the n memory capacity. In contrast, when t>n, window memory overflow occurs, the oldest memory $P_{i,t-n}$ is removed from the window memory container, and the recent memory $P_{i,t}$ is pushed to the end of the container. When corner reset is implemented and the point is tracked, the window memory container of the point is maintained, not reset.

(2) $$\Delta P_{i,t}=\sum_{t^{\prime }=t-n}^{t-1}\Delta P_{i,t^{\prime }}=\sum_{t^{\prime }=t-n}^{t-1}\left(P_{i,t^{\prime }+1}-P_{i,t^{\prime }}\right).$$

To enhance the detection performance, especially when capturing a distant moving object, the moving window method changes the distances of the first and last locations in the window memory, to determine whether the feature is a moving object. Instead of the sum of moving distances within a period, the threshold measurement is based on the varied distances of the points because the vibration related noise moves continuously in a certain period. In contrast to the summed value, the change in location in the period does not cumulate the moving distances, and only the changes in the initial and final point locations are determined. Thus, we define the change in point locations in a constant time interval, $\Delta P_{i,t}$, and use it instead of the sum of changes in the point locations *P*(*x*,*y*) from time t−n to t−1, as shown in Equation (2).

(3) $$L_{i,t}=dist\left(P{}_{i,t-n},P_{i,t}\right),\;D_{i,t}=\left\{\begin{array}{cc} 1, & \mathrm{if}\;L_{i,t}\geq \alpha \\ 0, & \mathrm{if}\;L_{i,t}<\alpha \end{array}\right..$$

In Equation (3), $L_{i,t}$ is the movement distance, measured using the Euclidean distance method, in units of pixels. Moreover, α is the distance threshold to determine whether the point is a noise or moving object. $D_{i,t}$ is a flag that operates the moving window detector function and identifies whether point $P_{i,t}$ is a moving target or vibration noise, based on $L_{i,t}$. For example, if $D_{i,t}=1$, the green detection box is generated around point $P_{i,t}$. A high distance threshold can block the sensor noise and obtain more definite movements, demonstrating lower recall but higher precision performance. The performance metrics must be adjusted based on the detection environments.

### 3.3. Memorized Estimator

**Figure 4:**
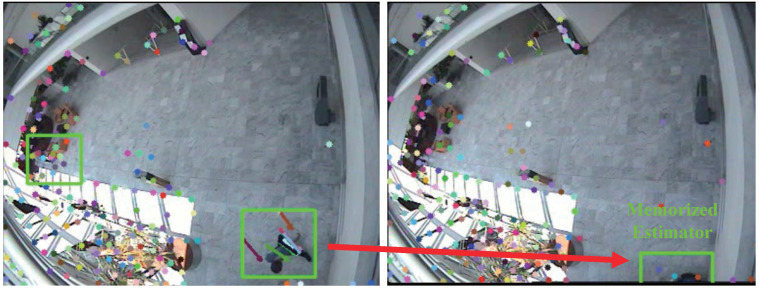
Example of the memorized estimator method. The green boxes show detection of moving objects. The green detected box is maintained on the target over a given time span.

We propose a function to estimate the location of the tracking object, even if it is temporarily stopped or hidden behind obstacles, by estimating the feature points pertaining to the target in Figure 4.

(4) $$E_{i,t}=\left\{\begin{array}{cc} \tau , & \mathrm{if}\;D_{i,t}=1\\ E_{i,t-1}-1, & \mathrm{if}\;D_{i,t}=0\;\mathrm{and}\;E_{i,t}>0. \end{array}\right.$$

In Equation (4), τ is the estimation time. $E_{i,t}$ represents the estimator for the feature point *i* at time *t* and is reset to τ when feature point *i* has a detection state of $D_{i,t}=1$, determined using Equation (3). If the moving window detector $D_{i,t}$ is set as zero and the detector $E_{i,t}$ is more than 0, the memorized detector $E_{i,t}$ is reduced to $E_{i,t}-1$ in each time step.

The non-zero estimator ($E_{i,t}>0$) attempts to detect hidden targets. For example, if certain feature points lose the tracking target or the target is hidden because it is beyond the camera frame or behind walls or obstacles, the memorized estimator continues to track the feature points of the target. The green detected box is maintained on the feature point while estimation time $E_{i,t}$. In addition to the effect of the moving window detector, a higher τ increases the recall and decreases the precision performance.

## 4. Experiments and Results

### 4.1. Experimental Environment

The proposed method is influenced by feature point characteristics such as the corners for calculating the optical flow and tracking the next point. Experiments are performed to identify an effective feature generating algorithm by comparing several algorithms. First, we specify the control parameters for all algorithms: corner reset interval, maximum number of corners and corner distance, semi-metric parameters, number of missing boxes (non-existent feature points in the label box), and number of real-generated features. To select the optimal parameters for each algorithm, we investigate the semi-metric comparison results, apply the selected parameters to the algorithms and evaluate the performance. The follow subsections describe the semi-metric parameters of each algorithm and performance evaluation.

#### 4.1.1. Dataset

**Figure 5:**
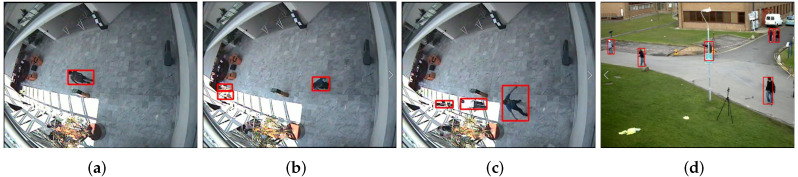
Examples of datasets (CAVIAR and PETS2009). (**a**): one person walks in a straight line (Walk). (**b**): people are browsing back and forth (Browse). (**c**): people are browsing while waiting (Browse_Whilewaiting). (**d**) Multiple pedestrians (pedestrian). The red boxes show the ground truth contained in the datasets.

We conduct an experiment by applying the proposed algorithm to two large datasets and perform a comparative analysis with other existing algorithms, as shown in Figure 5. Figure 5a–c show the first dataset: Videos 1, 2, and 3 correspond to walk data for a person walking in a straight line in a hallway, flow data for back and forth movement, and waiting data for a person pausing in the middle and then continuing to walk back and forth, respectively. The dataset has a resolution of 384 × 288, and the number of video frames are 790, 1042, and 610. The proposed algorithm is compared with dense and sparse optical flow algorithms. In the second dataset, video 4 shows the surroundings, containing more individuals than those in the first set. The resolution is 768 × 576, and the number of frames is 794. The proposed algorithm is compared with pedestrian detection algorithms (dense and sparse optical flow algorithms and Hog and Haar-like methods).

The first dataset focuses on the recognition and evaluation performance of moving objects instead of pedestrian shapes, and the second dataset is aimed at comparatively analysing the proposed algorithm with algorithms that can estimate shapes and moving objects. The two datasets are significantly different: The first dataset contains slowly walking people, whereas the second dataset contains rapidly walking people. In the first dataset, the moving targets are more difficult to detect because of the presence of fewer people and people who are walking slowly. Because the two datasets have different resolutions, we consider the resolutions of 384 × 288 and 768 × 576 as small scale and large scale, respectively, to ensure a fair comparison. In the comparison of the optical flow methods, video 4 is resized to the small scale ($\mathrm{video}\;4_{S}$, resolution 384 × 288). When comparing machine learning methods, videos 1–3 are resized to the large scale (videos 1–$3_{L}$, resolution 768 × 576).

#### 4.1.2. Missing Box of Corner Parameters

In the proposed method, to detect moving objects, we calculate the optical flows of each feature point, determine the next point movement location, and detect moving objects by inspecting the moving window memories of the points. Therefore, the feature points for calculating the optical flow and next movement are essential and important components of the proposed method. The feature points are typically spotted around existing moving objects and those that recently appeared in the frame. The feature points tracked on the moving objects remain on the target objects.

**Figure 6:**
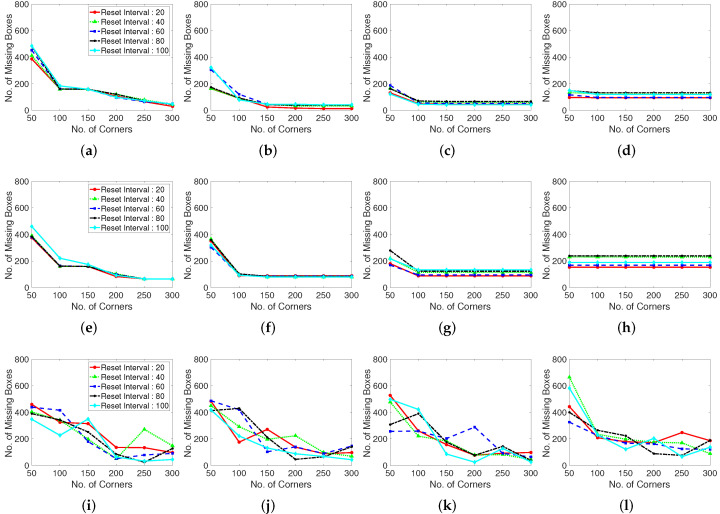
Number of missing boxes with various corner detection methods. The corner detection methods control the number of corner features and the pixel distance between corners. Test dataset is video 1, ‘Browse_WhileWaiting1.mpg’ from CAVIAR dataset. (**a**–**d**): Shi-Tomasi corner extraction (a pixel distance of 5, 10, 15, and 20 between corners). (**e**–**h**): Harris corner extraction (a pixel distance of 5, 10, 15, and 20 between corners). (**i**–**l**): Random grid point method (a pixel distance of 5, 10, 15, and 20 between corners).

To observe this situation and enhance the performance of the corner generation algorithm, we define a parameter, that is, the number of missing boxes, which counts the label boxes of non-existent feature points to determine the optimized values. In Figure 6, a higher number of missing boxes means that the feature point generation algorithm does not generate feature points to calculate optical flows. A lower number of missing boxes corresponds to a higher detection rate. When both number of generating corners and distance between corners low, corner features are generated densely, and it causes a lot of missing boxes.

**Figure 7:**
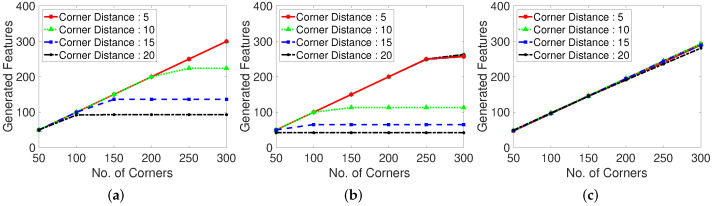
Generated features with various corner detection methods. Test dataset is video 1. (**a**): Shi-Tomasi corner extraction. (**b**): Harris corner extraction. (**c**): Random grid point method. Shi-Tomasi corner extraction tends to make more feature points than Harris corner extraction.

We specify the real generated corner numbers for the abovementioned experiment environments to examine the influence of the corner features on the number of corners, as shown in Figure 7. If the number of corners is large, the frames per second (FPS) for the processing is high. In contrast, if the number of corners is small, the accuracy of feature tracking to determine the optical flow are high. Certain feature point generating algorithms have dependent parameters such as the corner distance versus corner number. In the case of corner-generating algorithms, setting the corner distance limits the maximum number of generation points, depending on the inter-corner distance, maximum number of corners, and presence of corners in the frame. If the corner distance is excessively high, the number of corners is low at a given frame size. We examine the number of real generated corners or feature points for each feature generating method (Shi–Tomasi and Harris corner extraction algorithms and random grid point methods).

**Figure 8:**
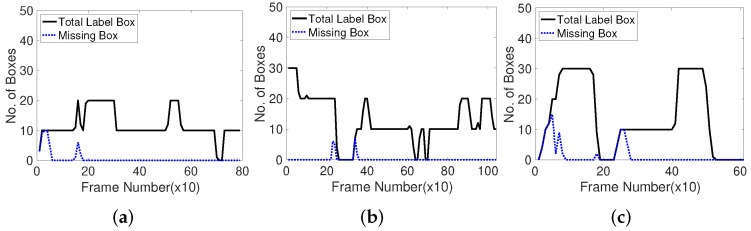
Number of missing boxes in every 10 frames with Shi-Tomasi corner detection method. Test datasets are videos 1, 2, 3. (**a**): Video 1 (Browse_Whilewaiting). (**b**): Video 2 (Browse). (**c**): Video 3 (Walk).

Considering the corner feature extraction results based on the number of missing boxes, the parameters of the corner-generating algorithms are determined to optimize the performance. To specify the best corner generation framework, we inspect the number of missing boxes in 10 frames of three typical public datasets. Figure 8 shows that the missing boxes are exposed at first, as indicated by the black solid line when the moving objects appear or disappear, and disposed off. This observation indicates that the feature points to track the moving objects are effective and appropriate.

#### 4.1.3. Evaluation of Corner Parameters

To evaluate the influence of the corner generation on the performance, we measure the recall and precision for the corner generation algorithms. The Shi–Tomasi and Harris corner extraction algorithms and randomly located point method are compared in terms of the LK optical flow tracking feature points. The variance in the corner generation parameters is the same as that in the previous subsection. In this section, the results of only one test dataset (Browse_WhileWaiting1.mpg) is presented owing to the limited space. The recall, precision, and number of missing boxes, and number of generated features are presented in the following figures.

The following metrics are typically used in object detection experiments: True positive (TP) means successful detection of the ground truth labels, false positive (FP) means detection failure, and false negative (FN) indicates the number of non-detected labels. The precision, recall, and F-score are determined as TP/(TP+FP), TP/(TP+FN), and 2×(Precision×Recall)/(Precision+Recall), respectively. In this case, β is 1. Precision indicates the detecting accuracy rate, recall represents the proportion of detected true labels, and the F-score is a generalized measurement considering both the recall and precision. These typical evaluation measurements are used in the following analyses.

**Figure 9:**
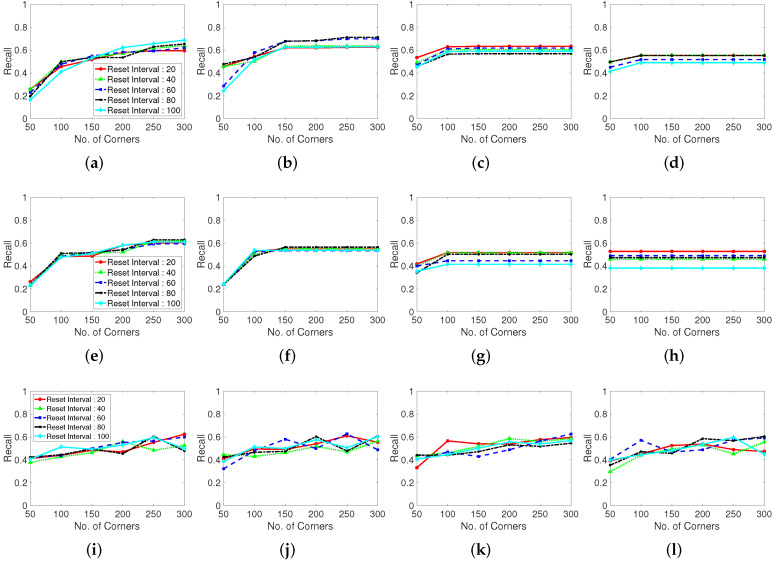
Recall performance with various corner detection methods. (**a**–**d**): Shi-Tomasi corner extraction (a pixel distance of 5, 10, 15, and 20 between corners), (**e**–**h**): Harris corner extraction (a pixel distance of 5, 10, 15, and 20 between corners) and (**i**–**l**): Random grid point method (a pixel distance of 5, 10, 15, and 20 between corners). Test dataset is video 1.

**Figure 10:**
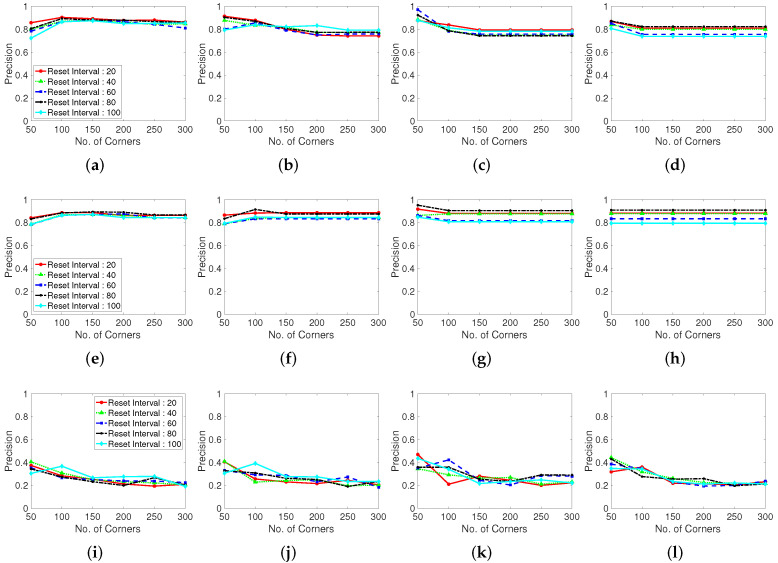
Precision performance with various corner-generating algorithms. (**a**–**d**): Shi-Tomasi corner extraction (a pixel distance of 5, 10, 15, and 20 between corners), (**e**–**h**): Harris corner extraction (a pixel distance of 5, 10, 15, and 20 between corners) and (**i**–**l**): Random grid point method (a pixel distance of 5, 10, 15, and 20 between corners). Test dataset is video 1.

Figure 9 shows the recall results for various corner parameters of the Shi–Tomasi corner extraction algorithm (first row), Harris corner extraction algorithm (second row), and randomly generated features (third row). With the increase in the maximum number of corners, the recall performance steadily increases and becomes convergent and stable. The Shi–Tomasi corner extraction algorithm outperforms the other algorithms in terms of the recall. As shown in Figure 10, the Shi–Tomasi and Harris corner extraction algorithms exhibit similar precision performances and the values converge, although the Shi–Tomasi algorithm is slightly superior. Thus, the most effective feature generating method is the Shi–Tomasi corner extraction algorithm with the maximum number of generated corners being 150–200. Corner reset interval parameters exhibit similar results over 60 frames. The corner distance parameters results are similar in four columns. We select the corner distance parameter as 10 pixels (second column), which corresponds to a stable and high performance in terms of the recall and precision. In the following analyses, we choose the best parameter values for the considered methods.

**Figure 11:**
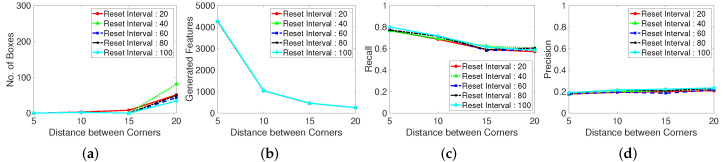
Performance with a regularly-spaced grid of sampling points. Test dataset is video 1. (**a**): Number of missing boxes. (**b**): Number of generated features. (**c**): Recall. (**d**): Precision.

Moreover, we evaluate the grid located point results. The randomly located point method exhibits an unstable performance, likely because of the stochastically generated point locations. Thus, well-distributed tracking point must be used when implementing a limited number of generation corner number. Distance between corner refers the parameter using in the corner extraction algorithm, which determines the distance between extracted corners. In the case shown in Figure 11, setting a maximum corner number is meaningless because the points are generated according to the grid of the constant corner distance. Therefore, we investigate the corner distance and corner reset interval parameters in terms of the number of missing boxes, number of real generated features, recall, and precision. The grid generated point method exhibits a large number of missing boxes, but the recall and precision are low. Moreover, the second plot in Figure 11 shows that the generation of excessively many tracking points decreases the computational speed.

### 4.2. Experimental Results and Comparisons

The proposed method differs from other moving-object detection algorithms owing to the implementation of the moving window and estimator method on the LK optical flow algorithm, which enhances the object detection performance and helps overcome the limitations of sparse optical flow techniques. The estimator operates synergistically with the moving window method by preventing the failure of determining the sparse optical flow. Specifically, when the moving window tracking for the change in the location of the feature points fails, the estimator is activated. The estimator remembers the last location of the tracking object and predicts the presence of the disappeared object on the spot. Therefore, we conduct experiments to examine the detection performance with changes in the parameters of the window and estimator: window sizes, distance thresholds, and estimation times.

We test four datasets: Browse_WhileWaiting1.mpg, Browse1.mpg, and Walk1.mpg from the CAVIAR dataset for comparing moving-object detection algorithms using typical sparse and dense optical flow; and PETS09-S2L1.webm from the ETS2009 dataset for comparing pedestrian detection algorithms with Hog and Haar-like SVM detection models [33,34]. The experiments for recall and precision are independent of the accuracy because this parameter is influenced by the algorithm parameter settings. Notably, FPS is influenced by the electric power stability of the device. Raspberry Pi 4 is used, and thus, 20 experiments are conducted, and the average and standard deviation of the 20 values are considered. We examine the effects of the moving window and estimator and compare the performance of other object detection algorithms.

#### 4.2.1. Results with Changes in Window Size

The window size is a key parameter of the moving window function in the proposed method. The function contains the locations of all tracking points (x,y) in the moving window memory from time t−windowsize to *t*, and thus, each window memory has a specific size. When the window memory is full, the last location memory is eliminated, and the recent location memory is pushed to the end of the list. Therefore, a constant window memory size is maintained. The window tracks the change in the location of each point and decides whether it is a moving object or noise by considering the moving distance threshold. The influence of the change in the window size is reflected in terms of the true number of detection boxes, recall, and precision. The total number of alarms is the total number of predictions obtained using the proposed detection method, and this value is compared with the true number of detection boxes.

In the case of a small window size, the history of the tracking point is limited, and the change in the points’ locations is observed. The probability of the point being identified as a noise instead of a moving object is higher. In contrast, for a large window size, the history of the tracking point is adequate, and the point location can be tracked to examine if it is moving object. However, the detection of true target boxes may be missed because considerable time is required for the evaluation, and the window may fail to track the point when the moving object disappears. For example, if the window size is 40 and the object is moving in 50 frames, the detection box has less than 10 frames. Therefore, the window size must be properly selected. We conduct experiments with different window sizes and examine their influence on the number of detected boxes and accuracy.

**Figure 12:**
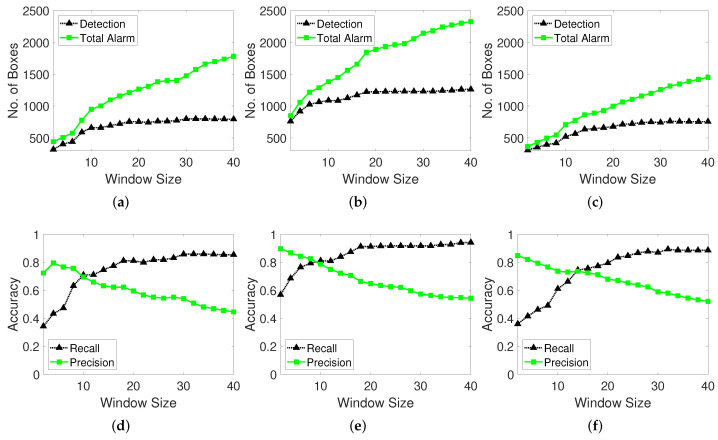
Number of boxes and accuracy performance of the moving window method with various window size parameters. Test datasets are video 1 for (**a**,**d**), video 2 for (**b**,**e**), and video 3 for (**c**,**f**). (**a**–**c**): Number of total predicted detections (total alarm) and true detections. (**d**–**f**): Recall and precision performance.

As shown in Figure 12, the number of detected moving object boxes increases with the window size, and the difference in the ratio of the total predicted detections and true detections increases. This gap signifies that as the window size increases, the proposed method tends to erroneously indicate that moving objects exist. In other words, extremely high window sizes deteriorate the detection performance because more time is required to decide whether the target is in a moving state. However, at larger window sizes, the number of true detected moving objects stabilizes but the number of prediction alarms increases. This aspect indicates that the abovementioned phenomenon likely has another explanation. When the algorithm examines a longer history of the tracking point and location changes (when the window size is larger), it is more likely to identify the target point as moving object even when it is stationary state. If the tracking point movement distance exceeds the moving window distance threshold, the moving window detection algorithm judges the object to be moving when the change in the location exceeds the threshold. Therefore, the object is likely to be predicted as a moving object even when the target tracking point stops. Sparse optical flow cannot easily track fast-moving objects and thus an object may be considered to be moving even when the tracking point does not lie on the moving object.

With the change in the window size, there occurs a crossing-over point at which the recall and precision curves intersect. This point is likely an optimal value to ensure a stable performance between the recall and precision as well as the F-score (F1). The total true detection number steadily increases and adversely influences the recall performance. Thus, longer tracking of the change in the point location leads to the detection of more true moving objects because the moving window detector obtains the interpretation based on a longer history at each point. The precision decreases as the recall increases because the optic flow tracking points that remain at and depart from moving object are considered to be in the moving state by the moving window memory. We validate this analysis by investigating the output detection labelled video. The red labelled box is the ground truth box, the green labelled box is the detection result of the moving-object detector algorithm, and the blue labelled box pertains to false detections. The findings indicate that the optimal window size is 10 frames.

#### 4.2.2. Results with Changes in Distance Thresholds

**Figure 13:**
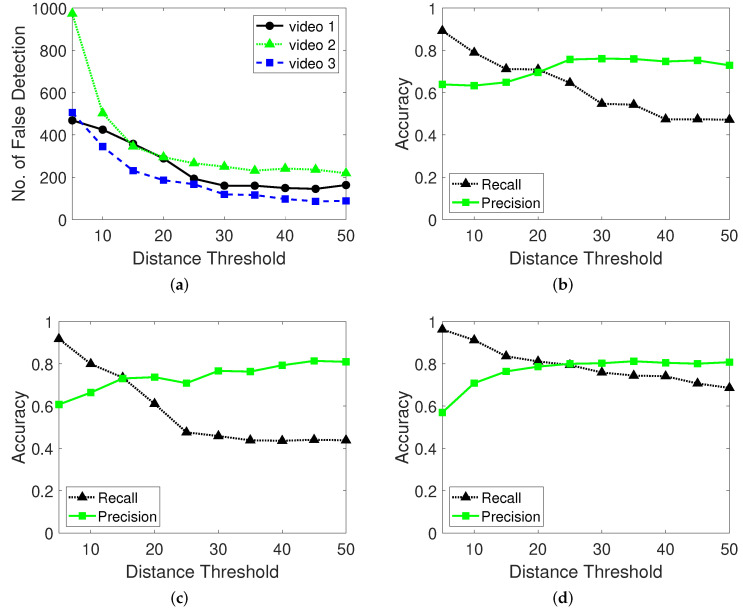
Number of false detections and accuracy performance of the moving window method with various distance threshold parameters. (**a**): Number of false detections for videos 1, 2, 3. (**b**): Recall and precision performance for video 1. (**c**): Recall and precision for video 2. (**d**): Recall and precision for video 3.

Figure 13 shows the number of false detections to validate the noise filtering effect based on the distance threshold in the moving window method. A high distance threshold prevents the detection of the noises from vibrating cameras in locations such as construction fields. In contrast, a low distance threshold enables the detection of minute noisy vibrations and small moving objects. A lower distance threshold corresponds to a higher recall and lower precision. The black line shows that high threshold distance filters the noises and tiny movements. The green line shows that noise filtering in the moving window distance threshold method helps achieve more precise results.

#### 4.2.3. Results with Changes in Estimation Time

The memorized estimator remembers the last position at which the calculation was stopped for a certain period (frames) and continues prediction even when the moving object stops or disappears from the video. To evaluate and select optimal parameters for this function, we determine the number of detections according to the estimation time and existence of the estimation function. The performance indicators of recall and precision are determined.

**Figure 14:**
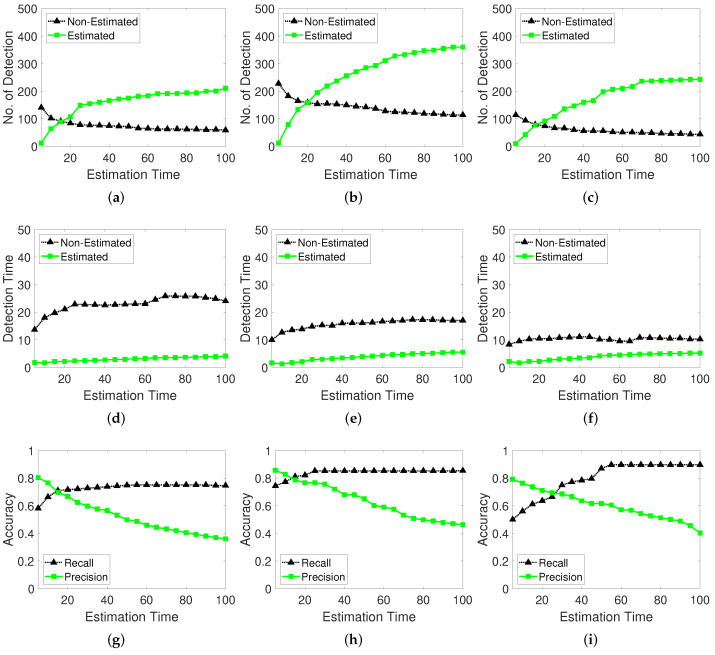
Estimation time of the memorized estimator method. Test datasets are video 1 for (**a**,**d**,**g**), video 2 for (**b**,**e**,**h**), and video 3 for (**c**,**f**,**i**). (**a**–**c**): Number of detections. (**d**–**f**): Tracking time with the memorized estimator. (**g**–**i**): Recall and precision performance.

Figure 14 shows the results of the number of detections, detection elapsed time, recall, and precision. When the proposed detector uses the estimator, the non-estimated detection number refers to the number of successful detections of the moving object. When the proposed detector uses only the moving window and distance threshold method, the estimated detection number means the detection counts when the detector estimates the target. The detection time is the elapsed time until the proposed detector identifies a moving object when working on an estimated or non-estimated tracking target. As shown in the first row of Figure 14, a larger tracking estimating time corresponds to a decreased probability of detecting moving objects than that pertaining to non-estimated tracking. When the estimation time is longer, the estimator spends more time on the moving targets and less time on the non-estimated targets because the detector for the moving objects implements the estimation more frequently. Moreover, the elapsed time until a moving object is detected is less than that for the non-estimated detection tracking. This finding shows that the estimator works faster when detecting moving objects if they temporarily stop, by carefully observing the movement.

The memorized estimator influences the detection accuracy. When the object is not sensitively detected, the detector misses the moving target and the estimator supplements the insufficient information of the moving target by memorizing the target’s last location information such as the moving window memory. As shown in the last row in Figure 14, the recall is stable as the estimation time increases higher; however, the precision decreases because the estimator remains activated when the moving target stops or moves behind obstacles such as walls, trees, or roofs, when observed through a top-view camera.

The proposed algorithm has three key parameters. Based on the experimental results, we determine the optimal parameters for the corner features (generation method, maximum corner number, and corner distance), memorized moving window (window size and distance threshold), and memorized estimator (estimation time).

**Figure 15:**
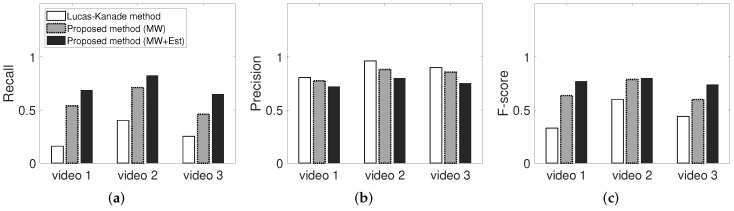
Performance (precision, recall, F-score) of proposed methods. Lucas–Kanade method is the original sparse optical flow, MW is the moving window without memorized estimator, and the moving window with memorized estimator. (**a**): Recall. (**b**): Precision. (**c**): F-score.

In Figure 15, LK method corresponds to the sparse optical flow, which is the basis of the proposed algorithm, MV corresponds to the proposed moving window detector without the memorized estimator, and MW + Est pertains to framework with the memorized moving window and memorized estimator. The recall and precision of the proposed algorithms (MV and MW + Est) are comparable to the existing algorithm. Thus, the proposed model exhibits a high accuracy and reproducibility for actual moving objects. Figure 15 shows the influence of the proposed methods (moving window detector and memorized estimator) on the recall and precision for three input videos.

The white bar corresponds to a low recall and high precision accuracy, which is not suitable for detection and may increase the possibilities of accidents pertaining to missing moving objects such as workers in the industrial field. The proposed moving window detector and memorized estimator can help enhance the safety and detection performance. Both methods exhibit higher recall and precision. Moreover, the estimator enhances the true detection rate, and the estimator influences the false detection rate for disappeared or stationary tracking objects. As shown in Figure 15c, the F-score slightly increases. In the industrial field, the safety of workers from dangers is more important than the false alarm rate. In particular, misdetections of workers operating near heavy equipment may lead to fatalities, whereas false alarms may simply be considered cautionary.

#### 4.2.4. Comparisons with Various Detection Algorithms

We halve or double the resolutions of the input videos according to the object detection algorithm. Machine learning detection algorithms such as Hog and Haar-like algorithms typically learn objects with resolutions of 64 × 128. The resolution of videos 1, 2, and 3 is 384 × 288 and that of video 4 is 768 × 576. The resolution of videos 1, 2, and 3 is converted to 768 × 576 and compared with video 4 in terms of machine learning object detection algorithms; the videos are named videos $1_{L}$, $2_{L}$ and $3_{L}$. Moreover, the resolution of video 4 is converted to 384 × 288 for optical flow object detection algorithms, and it is named video $\mathrm{video}\;4_{S}$.

The LK optical flow method using in the comparison experiment is a typical moving object detection algorithm based on sparse optical flow, and a simple noise filtering function of the moving distance threshold is applied between the locations of the previous and present pixel. The Farneback method is also a typical moving object detection algorithm based on dense optical flow and simple noise filtering function of the optical flow magnitude threshold for pixels. The Hog and Haar-like object detection algorithms are famous machine learning classification methods based on the learning weights of pedestrian. All the algorithm codes are sourced from the official OpenCV community site.

Our method consists of the moving window system and the target estimator. Although the computational speed of the proposed algorithm is similar to that of typical sparse optical flow in Table 1, as indicated by the FPS in the embedded system (Raspberry Pi 4), it achieves a higher recall and precision. Even at a slightly lower FPS, the proposed method outperforms the existing algorithm in terms of the recall, precision, and F-core. The dense method exhibits a low FPS but recall, precision, and F-score are similar to the proposed method, which performs extensive calculations for the optical flow vector and magnitude of all pixels. This finding indicates that the proposed method can optimize the costs of the applied functions (moving window detector and memorized estimator) and achieve a higher performance than the existing moving object detection algorithms based on the optical flow. In experiments on $\mathrm{video}\;4_{S}$, a lower FPS than other videos is achieved, and there are more moving objects in terms of the TP. The results for video 4 corresponds to a slightly increased recall but comparable precision. As mentioned, videos 1–3 have slow walking people, whereas video 4 has many people who walk rapidly. The proposed method exhibits a reasonable prediction performance in the presence of vibration noise. In the case of videos 1–3, the proposed method can effectively distinguish the slowly moving target and vibration noises. In contrast, the LK method evaluates slowly moving targets as vibration noises and thus cannot detect moving targets. In video 4, many pedestrians move rapidly, and thus, the LK method can effectively detect targets. In other words, the proposed method can robustly distinguish vibration noises and moving targets.

Table 2 indicates that the recall, precision, and FPS for the proposed algorithm are higher than the typical pedestrian classifiers, Hog and Haar-like methods. The Hog and Haar-like detection algorithms incur high calculation costs because they calculate the masks of the pixels and classify whether the pixels are objects from prebuilt learning weights. Notably, machine learning models cannot effectively detect objects that are distorted or rotated from those in the learning model. In the case of videos 1–$3_{L}$, the proposed method detects many moving objects, but the Hog and Haar-like methods miss the objects owing to distortion. In video 4, which is typically used for machine learning detection algorithm, the proposed method exhibits a comparable precision and higher recall than the compared algorithms. The findings indicate that different sizes and distortions of pedestrian objects affects the detection accuracy of Hog and Haar-like methods. In other words, the proposed method can outperform the machine learning algorithms in moving object detection.

Table 3 summarizes results for noisy video frames (blurred, poisson, gaussian, and salt-pepper noise in four videos), obtained using the proposed method and other methods. The considered noises are representative types of image noise. In the case of blurred noise, pixels in the blurred frame are filtered and averaged with five neighbouring pixels. Poisson noise is a type of electronic noise generated with the averaged distribution of extended scaling to the input pixel values. Gaussian noise is generated with gaussian distribution of zero mean and 0.01 variance. Salt-pepper noise is generated with a noise density of 0.05, which affects 5% of the pixels. Blurred and poisson datasets correspond to weak noise, and gaussian and salt-pepper datasets correspond to strong noise. Table 3 is presented in two parts: The upper table corresponds to weak noise, and bottom table corresponds to strong noise. The datasets include original noises from camera vibrations and image quality, and we add more intense noises to the datasets to verify the robustness of the methods against noise.

The Hog and Haar-like methods exhibit inferior detection performance and low robustness in various environments because of the fixed pretrained filter weight, distortions, and various sizes of the moving targets in the frame. We compare the LK and Farneback methods (as sparse and dense optical flow methods, respectively). The parameters of the compared methods is optimized to ensure a fair comparison. For the LK method, the corner quality parameter ranges from 0.01 to 0.1 to avoid a large number of corners being generated on the noise. For the Farneback method, we set the mean N parameter from 3 to 30 to normalize the baseline of decisions between noises and moving targets, which smoothens the generated optical flows. The precision, recall, F-score, and FPS results are compared.

The results presented in Table 3 are considered to evaluate the robustness of the proposed method. The precision, recall and F-score of the proposed method for videos 1, 2, 3, and $4_{S}$ is slightly deteriorated. The performance of the LK method is significantly deteriorated on the noisy datasets, especially in the case of salt-pepper noise. The LK method cannot effectively decide whether the feature points are noise or moving target owing to a large number of features generated on the noise. For the Farneback method, the recall is significantly decreased in the case of gaussian and salt-pepper noise videos. Because the Farneback optical flow is calculated on all pixels including noises, it can eliminate the noise effects, but loses the sensitivity of moving target detection. in the case of blurred noise, the Farneback method calculates the optical flow that is lower than that for normal datasets, resulting in slightly lower recall and higher precision. In contrast, the proposed and LK methods lose precision owing to the ambiguous generated corners on the blurred spot. The result of $\mathrm{video}\;4_{S}$ are similar for all methods in Table 1; however, in the cases shown in Table 3, the performance of the LK and Farneback methods are considerably different. This video has fast moving and many pedestrians without noises. However, the addition of blurred, gaussian, and salt-pepper noise renders $\mathrm{video}\;4_{S}$ challenging, more moving objects are detected as noise and vice versa.

The proposed method memorizes the location history of each feature point in the moving window and tracks the target in the window. The proposed method can effectively distinguish the additional noise and moving targets because each feature point has its own window memory. The proposed method outperforms the LK method and achieves a higher FPS than the Farneback method. Therefore, Table 3 demonstrates the robustness of the proposed method in noisy environments.

## 5. Conclusions

In this paper, a new approach to moving object detection and tracking is proposed. One of the major attributes is to improve the accuracy performance and reduce the computation time of responding to moving objects or moving pedestrians. The proposed method is based on the sparse optical flow approach, that is, a coarse-grained optical flow, but it includes the corner feature reset with a moving window. A sequence of images effectively finds the flow of moving objects and thus the moving window of image frames easily captures moving targets without wasting much time.

The moving window detector improves the noise filtering and the detection rate, by looking at a history of optical flows. In a hazardous environment, such as the construction sector, there may be the risk of meeting many obstacles including walls and trees, and various optical flow patterns are often observed, when pedestrians should be detected. The memory-based target estimator plays the role of monitoring the targets or pedestrians without missing when the targets move around or stagger at some positions. Even if a moving target is initially recognized, the target may move continuously with occasional pause. With this estimator, the last position of a moving targets is estimated and this improves the performance of detecting moving objects in a row.

We adapt this detection algorithm in the embedded board system, Raspberry Pi4, for real application. The experimental results demonstrate that the suggested approach is effective for preserving the detection performance even with a low computing power of the embedded device. According to the experimental results, our proposed method shows similar or higher accuracy performance, compared to the conventional algorithms for moving object detection using optical flows or vision processing algorithms: Lukas–Kanade’s method and Farneback’s method in addition to Hog and Haar-like methods. It also provides a more efficient computing time than dense optical flows and vision processing algorithms. The approach works well even for distorted views from a top-viewed camera and also for blurred images or noisy image frames, and thus it can be robustly applied to various environments. For future work, we consider the optical flow approach with the identification of targets. Deep learning approaches have been popular for identifying pedestrians or objects, but they need much computing time. The suggested approach helps to detect moving objects easily and then the objects could be identified with a small size of neural network to reduce the computing time.

## Figures and Tables

**Table 1:** Comparison results of the proposed method with object detection methods using optical flow; $\mathrm{video}\;4_{S}$ indicates a small scale of images from video 4.

Methods	Input	TP	FP	FN	Precision	Recall	F-score	FPS
Proposed Method	video 1	653	123	277	0.84	0.70	0.77	48.02 (±0.70)
	video 2	1038	207	303	0.83	0.77	0.80	45.31 (±0.50)
	video 3	595	156	258	0.79	0.70	0.74	46.36 (±0.93)
	$\mathrm{video}\;4_{S}$	4028	609	622	0.88	0.86	0.87	30.96 (±1.71)
Lucas–Kanade Method [31]	video 1	185	20	745	0.90	0.20	0.33	51.70 (±1.56)
	video 2	583	24	758	0.96	0.43	0.60	50.75 (±1.28)
	video 3	249	29	604	0.90	0.29	0.44	50.51 (±1.13)
	$\mathrm{video}\;4_{S}$	3736	476	914	0.89	0.80	0.84	36.62 (±1.49)
Farneback Method [30]	video 1	654	159	276	0.80	0.70	0.75	4.27 (±0.06)
	video 2	1041	264	300	0.80	0.78	0.79	4.29 (±0.05)
	video 3	563	112	290	0.83	0.66	0.74	4.23 (±0.05)
	$\mathrm{video}\;4_{S}$	3442	417	1208	0.89	0.74	0.81	4.23 (±0.06)

**Table 2:** Comparison results of the proposed method with other pedestrian detection methods (Hog and Haar-like); video $1_{L}$, $2_{L}$, $3_{L}$ indicate a large scale of images from videos 1, 2, 3.

Methods	Input	TP	FP	FN	Precision	Recall	F-score	FPS
Proposed Method	$\mathrm{video}\;1_{L}$	708	118	222	0.86	0.76	0.81	16.58 (±0.40)
	$\mathrm{video}\;2_{L}$	1076	208	265	0.84	0.80	0.82	16.43 (±0.28)
	$\mathrm{video}\;3_{L}$	645	119	208	0.84	0.76	0.80	16.61 (±0.29)
	video 4	4117	440	533	0.90	0.89	0.89	12.20 (±0.09)
Pedestrian Detector (HOG) [33]	${}_{L}$	5	2	925	0.71	0.01	0.01	1.29 (±0.01)
	$\mathrm{video}\;2_{L}$	40	37	1301	0.52	0.03	0.06	1.29 (±0.01)
	$\mathrm{video}\;3_{L}$	2	2	851	0.50	0.001	0.001	1.28 (±0.02)
	video 4	2886	63	1764	0.98	0.62	0.76	1.29 (±0.01)
Pedestrian Detector (Haar-like) [34]	${}_{L}$	6	1	924	0.86	0.01	0.01	2.04 (±0.02)
	$\mathrm{video}\;2_{L}$	22	0	1319	1.00	0.02	0.03	2.00 (±0.02)
	$\mathrm{video}\;3_{L}$	1	10	852	0.09	0.001	0.001	1.96 (±0.03)
	video 4	2912	341	1738	0.90	0.63	0.74	1.83 (±0.02)

**Table 3:** Test results with blurred, poisson, gaussian and salt-pepper noises.

Methods	Input	Blurred				Poisson			
		Precision	Recall	F-score	FPS	Precision	Recall	F-score	FPS
Proposed Method	video 1	0.68	0.65	0.67	48.54	0.80	0.59	0.68	47.81
	video 2	0.83	0.75	0.79	45.34	0.80	0.72	0.76	45.37
	video 3	0.78	0.57	0.66	45.76	0.72	0.64	0.68	45.10
	$\mathrm{video}\;4_{S}$	0.86	0.88	0.87	31.43	0.89	0.85	0.87	31.27
Lucas–Kanade Method [31]	video 1	0.55	0.14	0.23	51.12	0.74	0.17	0.27	51.09
	video 2	0.82	0.33	0.47	51.34	0.75	0.36	0.49	50.51
	video 3	0.64	0.26	0.37	50.87	0.59	0.28	0.38	49.96
	$\mathrm{video}\;4_{S}$	0.92	0.58	0.71	36.49	0.90	0.52	0.66	35.88
Farneback Method [30]	video 1	0.86	0.65	0.74	4.27	0.63	0.64	0.64	4.26
	video 2	0.86	0.73	0.79	4.30	0.86	0.70	0.77	4.29
	video 3	0.96	0.58	0.73	4.22	0.89	0.52	0.65	4.21
	$\mathrm{video}\;4_{S}$	0.90	0.74	0.81	4.21	0.86	0.70	0.77	4.21
**Methods**	**Input**	**Gaussian**				**Salt & Pepper**			
		**Precision**	**Recall**	**F-score**	**FPS**	**Precision**	**Recall**	**F-score**	**FPS**
Proposed Method	video 1	0.59	0.52	0.55	47.29	0.63	0.43	0.51	46.18
	video 2	0.68	0.69	0.68	44.65	0.64	0.54	0.58	43.46
	video 3	0.65	0.54	0.59	44.76	0.52	0.53	0.52	44.20
	$\mathrm{video}\;4_{S}$	0.88	0.77	0.82	30.21	0.86	0.69	0.77	29.43
Lucas–Kanade Method [31]	video 1	0.40	0.13	0.20	50.13	0.29	0.07	0.11	48.56
	video 2	0.72	0.25	0.37	49.87	0.33	0.14	0.20	46.93
	video 3	0.57	0.22	0.32	49.01	0.30	0.12	0.17	47.04
	$\mathrm{video}\;4_{S}$	0.91	0.47	0.62	35.19	0.80	0.37	0.50	33.85
Farneback Method [30]	video 1	0.70	0.32	0.44	4.18	0.51	0.31	0.38	3.63
	video 2	0.88	0.49	0.63	4.19	0.78	0.48	0.60	3.74
	video 3	0.83	0.35	0.49	4.17	0.56	0.35	0.43	3.60
	$\mathrm{video}\;4_{S}$	0.76	0.63	0.69	4.04	0.73	0.63	0.68	3.37

## Data Availability

Not applicable.
